# Antimicrobial Resistance in the Context of the Sustainable Development Goals: A Brief Review

**DOI:** 10.3390/ejihpe11010006

**Published:** 2021-01-19

**Authors:** Márió Gajdács, Edit Urbán, Anette Stájer, Zoltán Baráth

**Affiliations:** 1 Department of Pharmacodynamics and Biopharmacy, Faculty of Pharmacy, University of Szeged, Eötvös utca 6., 6720 Szeged, Hungary; 2 Institute of Medical Microbiology, Faculty of Medicine, Semmelweis University, Nagyvárad tér 4., 1089 Budapest, Hungary; 3 Department of Medical Microbiology and Immunology, University of Pécs Medical School, Szigeti út 12., 7624 Pécs, Hungary; urban.edit@pte.hu; 4 Institute of Translational Medicine, University of Pécs Medical School, Szigeti út 12., 7624 Pécs, Hungary; 5 Department of Periodontology, Faculty of Dentistry, University of Szeged, Tisza Lajos körút 62–64., 6720 Szeged, Hungary; stajer.anette@stoma.szote.u-szeged.hu; 6 Department of Prosthodontics, Faculty of Dentistry, University of Szeged, Tisza Lajos körút 62-64., 6720 Szeged, Hungary; barath.zoltan@stoma.szote.u-szeged.hu

**Keywords:** antibiotics, antibiotic resistance, MDR, Sustainable Development Goals (SDGs), poverty, global health, health policy, COVID-19

## Abstract

The reduction in infectious disease morbidity and mortality may be attributed to a variety of factors; however, improved sanitation and public health, and the introduction of vaccines and antibiotics are among the most significant. The development of antimicrobial resistance (AMR) in bacterial pathogens is an expected consequence of evolutionary adaptation to these noxious agents and the widespread use of these drugs has significantly sped up this process. Infections caused by multidrug resistant pathogens are directly associated with worse clinical outcomes, longer hospital stays, excess mortality in the affected patients and an increasing burden and costs on the healthcare infrastructure. The Sustainable Development Goals (SDGs) were published in 2015 by the United Nations to serve as a global blueprint for a better, more equitable, more sustainable life on our planet. The SDGs contextualize AMR as a global public health and societal issue; in addition, the continuing emergence of AMR may limit the attainment on many SDGs. The aim of this mini-review is to provide insight on the interface between attainment of SDGs and the clinical problem of drug resistance in bacteria.

## 1. Introduction

Since the occurrence of the so-called epidemiological transition, infectious diseases and pandemics—which have previously decimated the population—have started to recede, while the life-expectancy of people around the world has increased significantly [1]; although HIV/AIDS, tuberculosis (caused by the bacterium *Mycobacterium tuberculosis*), diarrheal illness and neglected tropical diseases (NTDs) were still major causes of suffering in developing countries, for a short while, it seems that humanity have finally conquered infectious diseases [2]. Consequently, the structure of the disease burden has shifted considerably to civilizational illnesses, with cardiovascular illnesses, cancer and degenerative diseases becoming the leading causes of death [3]. The reduction in infectious disease morbidity and mortality may be attributed to a variety of factors; however, improved sanitation and public health, the introduction of vaccines and antibiotics are among the most significant [4]. The discovery and subsequent clinical use of antibiotics may be considered as one of the game-changing achievements in medicine, revolutionizing the care of patients, who would have previously succumbed to the onslaught of common or deadly bacterial infections, including (in decreasing incidence) respiratory tract infections, gastro-intestinal infections, urinary tract infections, skin and soft tissue infections, bacteremia and others [5,6]. Since the 1950s, antibiotics have saved millions of lives (both immunocompetent and immunocompromised patients) and they have allowed for the development of complex medical interventions and specialties, which would not have been previously possible [7]. However, the emergence of bacteria resistant to these drugs has proven to be one of the most serious concerns of the millennia.

The development of antimicrobial resistance (AMR) in bacterial pathogens is an expected consequence of evolutionary adaptation to these noxious agents [8]; nonetheless, the widespread use of these drugs has significantly sped up this process [9]. Bacteria can evade the effects of antibiotics in a multitude of ways, including the production of degrading enzymes (e.g., β-lactamases), adaptation to alternative metabolic pathways (e.g., folic acid metabolism), target alteration (e.g., modifications in ribosomal subunits or the topoisomerase enzymes), deceased uptake of the drugs (e.g., outer membrane protein mutants), over-expression of efflux pumps or by the production of a protective exopolysaccharide matrix or biofilm [10,11]. The susceptibility of these bacterial pathogens is assessed in clinical microbiology laboratories using conventional/phenotypic (e.g., disk diffusion, E-tests, broth microdilution, automated systems) and genotypic tests (i.e., identification of the carriage of resistance-determinants) [12,13,14]. Multidrug-resistant (MDR) bacteria microorganisms can withstand previously lethal doses of structurally and pharmacologically distinct antibiotic groups and persist in vivo: MDR is usually defined as bacteria resistant to three of more different antibiotic groups; these extensively drug-resistant (XDR) pathogens are only susceptible to two remaining agents, while pandrug-resistant (PDR; or totally-drug resistant, TDR) bacteria show non-susceptibility to all relevant antibiotics [15,16]. The increasing number of reports in the literature regarding PDR bacteria is the clinical practice in concerning for everyone [17,18]. Based on their overall clinical impact and significance, the so-called “ESKAPE” pathogens (E: *Enterococcus faecium*, S: *Staphylococcus aureus* or recently *Stenotrophomonas maltophilia*, K: *Klebsiella pneumoniae* or recently C: *Clostridioides difficile*, A: *Acinetobacter baumannii*, P: *Pseudomonas aeruginosa*, E: *Enterobacter* spp., or recently *Enterobacteriaceae*) receive the most attention, both from public health authorities and from drug development agencies [19]. The Centers for Disease Control and Prevention (CDC) in the US also published a classification of the most concerning AMR threats, where they categorized carbapenem-resistant *P. aeruginosa* and *A. baumannii*, *C. difficile*, *MDR Neisseria gonorrhoeae* and carbapenem- and cephalosporin-resistant *Enterobacteriaceae* as “urgent” threats [20]. The latter has been further highlighted by the Organization for Economic Co-operation and Development (OECD) report, calling attention to the global spread of carbapenem-resistance [21]. In 2017, the World Health Organization (WHO) has also put forth a list of “priority” pathogens, to serve as a guide and to prioritize antimicrobial research and development (R&D) to various Gram-negative bacteria (including non-fermenters and members of the *Enterobacteriaceae*), posing as particular threats due to their pathogenic potential and transmissibility (e.g., in nosocomial environments or nursing homes) [22].

## 2. Clinical Significance and Driving Forces behind AMR

As previously stated, microorganisms possess a plethora of mechanisms—which are intrinsic to the bacterial genus or acquired from exogenous sources (encoded by antibiotic-resistance genes (ARGs) to enhance their survival [23,24]. The emergence and spread of ARGs may be mediated by spontaneous mutations and subsequent vertical transmission (i.e. the progeny cells possess the resistance determinant originally “developed” by the original bacterial cells and via inter- and intra-species horizontal gene transfer (HGT); the significance of the latter is much higher, as many different resistance-determinants may reside on various mobile genetic elements (plasmids, transposons and integrons) with the ability to spread worldwide and among different bacterial genera [25,26]. The importance of HGT in AMR was highlighted by the rapid global spread of extended-spectrum β-lactamases (ESBLs) in *Enterobacteriaceae* [27]. More recently, the emergence and spread of carbapenemases in Gram-negative has been identified as a critical public health issue (at least partly owing to their extensive use for the treatment of infections caused by ESBLs); while chromosomal carbapenemases were described previously, their detection on mobile genetic elements allowed for their widespread dissemination (with the New Delhi metallo-β-lactamase 1 (NDM-1, first detected in India) being a pivotal example) [28]. Based on their substrate and inhibitor-profiles, carbapenemases may be differentiated via the Ambler-classification as serine-type (Class A: KPC (*K. pneumoniae* carbapenemase), Class D: OXA (oxacillinase)) and metallo-β-lactamases (Class B: IMP (imipenemase), VIM (Verona integron-borne metallo-β-lactamase) and NDM) [29,30]. Subsequently, carbapenem-resistance has forced the hand of clinicians around the globe to utilize polymyxins as last-resort drugs in resistant infections, leading to the description of the Mobilized Colistin Resistance (MCR-1, first detected in China) gene, signaling the emergence of plasmid-borne resistance to last-resort antibiotics [31,32].

Two phenomena have been identified as the main driving forces behind the clinical problem of antibacterial resistance in human medicine: on one hand, the imprudent use of these agents (including overuse (administration of antibiotics in inappropriate indications) and misuse (administration of antibiotics in inappropriate doses and durations)), which facilitates the development of resistance in bacteria (this is why these drugs are often termed “social medicines”, because the misuse by one person affects the efficacy of these drugs for society as a whole) [33,34,35]; on the other hand, pharmaceutical companies are turning away from the marketing authorization of novel antimicrobial drugs, due to the difficulties in drug development, the lack of returning financial investments and the inevitable emergence of resistant strains [36]. This is exemplified by the fact that no new broad-spectrum agent with a novel mechanism of action received marketing authorization since the introduction of the fluoroquinolones in the 1980s [37]. Unavailability of primary care (a good indication of a country’s healthcare system), poor public health conditions (corresponding to high incidence of infectious diseases), over-the-counter sales of antimicrobials and the lack of health literacy in patients, these medications (especially if they were obtained without a prescription) are all facilitators of AMR [38]. In a continent-wide survey sequestered by the European Commission in 2018, the Special Eurobarometer Report 478 has found that 32% of respondents have taken antibiotics per os in the last 12 months (showing an increasing prevalence in a gradient of west-to-east and north-to-south throughout the continent); overall, 7% of antibiotics were taken without a prescription (including non-prescription sales from pharmacies, leftover drugs or antibiotics received from friends/family). Symptoms and inappropriate conditions, such as a cold, flu or sore throat were reported as the reason for taking antibiotics in almost 40% of cases; in addition, 28% of respondents in the EU thinks that antibiotics are effective against the cold, while 48% believe that these drugs are useful in viral infections [39].

Self-medication with antibiotics (SMA) is defined as the use of these agents on the basis of the individual’s symptoms (without any consultation with a physician or a diagnosis), with the aim of alleviating these complaints [40]. The prevalence of SMA has been reported to be between 7.3–81.3% in low- and middle-income countries (LMIC) and 1–66% in high-income countries, based on two recently published scoping reviews [34,41]. In both studies, avoiding losing working days and employment was reported as one of the main reasons for SMA. In both geographical regions, the most commonly taken drugs for SMA are β-lactam antibiotics (i.e., amoxicillin, ampicillin), however, there are pronounced differences seen in the sources and reasons for SMA [34,41]. In developed countries, the main source of these drugs were leftover antibiotics, family members/friends, internet shops and veterinary pharmacies (i.e., non-prescription sales through community pharmacies are less common), while in many developed countries, antibiotics are available over-the-counter in pharmacies and from informal healthcare providers. Unsurprisingly, SMA is more commonly seen in developed countries, where this is usually coupled with the unavailability of physicians [34,41]. In the US, SMA is frequent in immigrants and people without health insurance [41]. Healthcare professionals (HCPs), including medical doctors, dentists, pharmacists and prescribing nurses have all pivotal roles as “gate-keepers” ensuring that these medicines are only used when necessary, while non-prescribing HCPs may also have roles in patient education [42]. In fact, based on the estimations of the European Surveillance of Antimicrobial Consumption Network (ESAC-Net) and the CDC, the majority of antibiotic use is unnecessary in some illnesses managed in the primary care settings (Table 1) [43]. For some infections (e.g., urinary tract infections), the presence of symptoms are good indicator for the need of antibiotic-administration; on the other hand, upper respiratory tract infections are, for the most part, of viral origin (i.e., influenza virus, parainfluenza virus, rhinovirus, respiratory syncytial virus, adenovirus), where antibiotics should not be administered without the clarification of the bacterial etiologies, e.g., with culture results or rapid tests (like Strep A rapid antigen assay) [44]. For this reason, the WHO has published a competency framework for all HCPs, defining knowledge-levels and attitudes to successfully combat AMR in everyday practice [45]. To improve the understanding of the general public regarding antibiotics and the dangers of AMR, the WHO has introduced the World Antibiotic Awareness Week (18–24 of November), while the European Centers for Disease Prevention and Control (ECDC) introduced the European Antibiotic Awareness Day (18th of November) [46]. While the continuous surveillance, policy changes and governmental restrictions had positive effects on the judicious use of antibiotics in Western countries, the same positive trend cannot be observed in many developing countries in Asia or Africa [47].

Infections caused by MDR pathogens are directly associated with worse clinical outcomes, longer hospital stays, excess mortality in the affected patients and an increasing burden and cost on the healthcare infrastructure [48,49]; the direct and indirect consequences of AMR are summarized in Table 2. Many national and international bodies’ estimations and projections on the incidence and consequences of MDR infections have declared for the government stakeholders around the globe to take action. According to present-day data, around 700,000 people die from bacterial infections caused by drug-resistant bacteria [50]. Based on the ECDC, MDR bacteria are responsible for over 400,000 infections and 25,000 excess deaths annually [51], while the CDC estimated over two million MDR infections and 23,000 excess deaths per year [52]. Similar projections have been also carried out for Thailand (25,000 deaths/year) and India (38,000 deaths/year) [53,54]. In the European Union and European Economic Area alone, there have been over 700,000 MDR infections, 33,110 excess deaths and around 875,000 disability-adjusted life years (DALY) reported by the Burden of Antimicrobial Resistance Collaborative Group for 2015 [55]. The World Economic Forum (WEF) has compared the insidious nature of the resistance problem to that of climate change [56]. The O’Neill Report—sequestered by the UK National Health Service—projected the worse outcome for humanity and global health, in the context of AMR: financial costs of 100 billion US dollars and over 10 million excess deaths, associated with drug resistance by 2050 [57]. The expected mortality rates will impact continents differently: ~500,000 deaths are expected on the European continents, while a much larger toll might be seen in Africa (~4 million) and Asia (~4.5 million) [57]. The concern for antibiotic resistance has been highlighted by the fact that the issue has been discussed by the United Nations (UN) General Assembly; this was only the fourth time a health-related issue has even been considered by the UN [58].

In the recent years, there has been a fundamental understanding that the issue of AMR needs to be addressed using a One Health approach, which—apart from the use of these drugs in human medicine—also takes into consideration the considerable use of antibiotics in animal husbandry, the presence of antimicrobials in food products and in the environment (e.g., water, sewage from hospitals or animal farms) through anthropogenic contamination [59,60]. In line with this, the design and implementation of interdisciplinary interventions need to be done to address AMR-related issues [61]. The majority of global antibiotic consumption is attributable to their use in animal husbandry (both as preventative medication and as growth promoters for livestock) and in veterinary medicine (for companion animals); around 70–80% of antibiotic-consumption is attributed to these industries [62,63]. Livestock production is an important aspect of many national economies (in the context of gross domestic product, food, raw materials and agriculture operations). Previously, the unrestricted use of antimicrobials (e.g., avoparcin) was common with the aim of maximizing profits, which has led to catastrophic consequences; therefore, prohibition provisions were devised in many countries to restrict the administration of antibiotics to cases only when it is medically indicated [64,65].

## 3. Sustainable Development Goals (SDGs), Interface between AMR and SDGs

The Sustainable Development Goals (SDGs) were published in 2015 by the UN within the program of “2030 Agenda for Sustainable Development” to serve as a global blueprint for a better, more equitable, more sustainable life on our planet [66]. The UN Earth Summit (Rio de Janeiro, 1992) and the UN Millennium Summit (New York, 2000) resulted in the antecedents of SDGs, the Agenda 21 and the Millennium Development Goals (MDGs), respectively [67,68]. The SDG initiative includes 17 well-defined goals from the fields of ecology, climate change, societal issues, economy, education and healthcare—that are frequently interlinked—with well-defined actions, targets and monitoring criteria to allow for the evaluation of the progress of these goals (Table 3) [69]. These goals were universally adopted by all UN member states, with the SDGs being continuously followed-up and reviewed by High-level Political Forum on Sustainable Development, seating annually. The deadline for attaining most of the SDGs has been set in the year 2030; however, others do not have a specific deadline [69].

Unsurprisingly, increasing levels of AMR threaten the attainment of the SDGs as this phenomenon considerably influences changes in society and healthcare: it can be said that the SDGs contextualize AMR as a global public health and societal issue [70]. This may have been worsened by the onset of the global severe acute respiratory syndrome coronavirus 2 (SARS-CoV-2) pandemic, which has not only further substantiated societal inequalities and economic difficulties, but has also led to a considerable increase in antibiotic use worldwide for the treatment of patients; it is not yet known what will be the long-term repercussions of this pandemic in the context of SDGs [71,72]. Most notably, the use of antibiotics has increased substantially in intensive care units (therapeutically and prophylactically) in the management of patients suffering from coronavirus disease (COVID-19) [73]. Antibiotic consumption in Hungary (before the onset of the pandemic) may be considered as appropriate from a quantitative standpoint (per capita consumption), however, qualitatively (characterized by the ratio of broad spectrum/narrow spectrum agents used), this country performs among the worst in the EU, mostly owing to the high rate of fluoroquinolone consumption [43]. In addition, a study has highlighted that the unavailability of general practitioners in various geographical regions of Hungary also leads to worse antimicrobial use qualitatively [74]. Among other antibiotics, the use of azithromycin has received substantial attention in COVID-19; while azithromycin has been known to have potent anti-inflammatory properties in the lungs (this has been described in patients with cystic fibrosis), some early studies on COVID-patients have also suggested this antibiotic as a potential treatment option with direct effects on the virus; however, the clinical evidence on this subject is highly controversial [75,76,77]. Nevertheless, azithromycin has been included in many institutional and local treatment protocols (even though unnecessary use of this drug may also bring forth serious cardiovascular adverse events), both in Hungary and elsewhere around the world [75,76,77]. This will inevitably lead to the selection of resistant mutants in bacteria (e.g., respiratory tract pathogens, atypical bacteria) where azithromycin has therapeutic relevance, which may hinder appropriate therapy for future patients [78]. It must also be mentioned that many other antimicrobial agents (e.g., hydroxychloroquine, ivermectin) were also proposed in the therapy of COVID-19, which has led to widespread attempts to procure these drugs by laypeople, going as far as looking for them in veterinary shops or pharmacies [79].

Among other things, the continuing emergence of AMR may limit the attainment of Goal 1 (No Poverty) and 2 (Zero Hunger). Due to the increasing global population, economic growth and developments in consumption habits, food production is expected to grow by 50–70% in the timeframe between 2010–2030; in parallel, the use of antimicrobials in food production (e.g., meat, milk, eggs) is also expected to increase by a similar margin [80]. This has been already demonstrated by major reports: while the increase in global antibiotic consumption in human medicine only increased by ~10%, consumption of these drugs in animal husbandry increased by almost 180% in the “BRICS” conglomerate countries (Brazil, Russia, India, China, and South Africa), with continuous increasing trends expected till 2030 [81,82]. Resistant bacteria threaten long-term food security, in addition, they may lead to the demise of the economic prospects of farmers (see Goal 8: Decent Work and Economic Growth). The same thing may be pointed out for people experiencing adverse outcomes after MDR infections, which may affect their opportunities to find employment and to perform at their workplace. People living in poverty are generally more vulnerable to infectious diseases and they are at a bigger risk to being affected by drug- resistant bacteria; these patients often do not have the means to obtain some of the more expensive medications and this is one of the reasons why self-medication with antibiotics is so common in regions with less developed infrastructures [83,84]. It may be said that antibiotic resistance can directly worsen societal inequalities (Goal 1 and Goal 10: Reduce Inequalities). Conversely, implementation of Goal 6 (Clean Water and Sanitation) will hopefully curb the need for antibiotics by reducing the prevalence of several infections (i.e., bacteria affecting the gastro-intestinal tract), because as many as 200 diseases may be transmitted by contaminated water, and it may also reduce the spread of MDR (e.g., from hospitals or animal farms) to the environment.

The relationship between AMR and worsening climate change is a discreet, but an important one, nevertheless, Goal 13 (*Climate Action*). In fact, in the report by the WEF, the similarities of climate change and AMR are shown as being insidious, inter-disciplinary and urgent problems for humanity [56]. Changes in the global climate will undoubtedly result in alterations in the diversity and complexity of microbial populations around the world; although the emergence of novel bacterial diseases is unlikely, the number of people at risk for many of these infections will undoubtedly increase, including vector-borne and zoonotic pathogens, and illnesses associated with food [85,86]. A 1 °C increase in environmental temperatures may be responsible to an increase of 5–10% in the number of foodborne salmonellosis cases, leading to substantial healthcare and economic costs [87]. It has also been described that increasing temperatures may favor the selection of resistant isolates [88]. Antibiotics should be considered important hallmarks of present-day medicine; thus, it is unsurprising that the Goal 3 (Good Health and Well-Being for All at All Ages) will never be achieved if the disadvantageous developments in resistance and the associated excess death toll are not addressed. Goal 12 (Responsible Consumption and Production) may also be relevant from the standpoint of facilitating prudent antibiotic use and antimicrobial stewardship; on the other hand, it is also in the best interest of governmental and supra-governmental organizations to advance public–private partnerships with pharmaceutical companies and biotechnological firms interested in getting involved in antimicrobial research [89,90]. As resistant bacterial isolates (and AMR in general) do not respect borders, the long-term management of resistance may only be successful with the full and continuous collaboration of all global stakeholders and countries (Goal 17: Partnership for the Goals), which is also an intrinsic requirement for the success of the SDGs. In addition to the many international declarations (listed in Section 2), in 2017, health ministers of the G20 countries have made a historic declaration to fight AMR on a global scale in a three-pronged approach (including conservation, responsible access and antimicrobial innovation) [91].

## 4. Concluding Remarks

By the beginning of the 21st century, it has become painfully clear that AMR has become one of the major issues in human medicine. Previously, we have highlighted the multilateral and inter-disciplinary interface between SDGs and AMR. To monitor the impact of antibiotic resistance and to track the progress of specific interventions—in the context of SDGs—an important issue that needs to be addressed is the development of a specific indicator for AMR, or for the hindrance AMR causes in the attainment of SDGs [69]. As previously mentioned, most of the SDGs have clearly-defined performance indicators and specific actions to attain the set goals. Some suggest that ensuring universal health coverage globally would widen the scope of visibility for sustainable development [92]. The more one assesses the importance of having functional primary healthcare and effective antibiotics available, the inter-relatedness of resistance and the SDGs becomes more apparent [93]; these common points should be highlighted for government stakeholders to facilitate the act of taking on the fight against AMR into national agendas.

## Figures and Tables

**Table 1:** Relevance of antibiotic-therapy in common ailments managed by primary care [43].

	Upper Respiratory Tract Infections (in Patients Aged <1 Year)	Acute Tonsilitis (in Patients Aged <1 Year)	Acute/Chronic Sinusitis (in Patients Aged ≤18 Years)	Acute Otitis Media (in Patients Aged <2 Years)	Acute Bronchitis/Bronchiolitis (in Patients Aged 18–75 Years)	Pneumonia (in Patients Aged 18–75 Years)	Acute Cystitis (in Female Patients Aged ≤18 Years)
Antibiotic therapy is indicated (based on ESAC */CDC * estimations)	0–20%	0-20%	0–20%	0–20%	0–30%	90–100%	80–100%

* ESAC: European Surveillance of Antimicrobial Consumption Network (ESAC-Net); CDC: US Centers for Disease Control.

**Table 2:** Direct and indirect consequences of AMR (antimicrobial resistance).

Direct	Indirect/Inability to Perform:
Decreased efficacy of available antimicrobial drugs	Complex surgical interventions
The onset of administering	Organ transplantation
The recovery rate and quality of life (QoL) of affected patients decreases	Cancer chemotherapy
Healthcare use and length of hospital stay increases	Intensitve care
Increased costs for the healthcare infrastructure	Neonatology
Decreased trust in medicine and pharmaceuticals	

**Table 3:** The list of UN Sustainable Development Goals (2015–2030) *.

**Goal 1: No Poverty**
**Goal 2: Zero Hunger**
**Goal 3: Good Health and Well-Being**
Goal 4: Quality Education
Goal 5: Gender equality
**Goal 6: Clean Water in Sanitation**
Goal 7: Affordable and Clean Energy
**Goal 8: Decent Work and Economic Growth**
Goal 9: Industry, Innovation and Infrastructure
**Goal 10: Reduced Inequalities**
Goal 11: Sustainable Cities and Communities
**Goal 12: Responsible Consumption and Production**
**Goal 13: Climate Action**
Goal 14: Live Below Water
Goal 15: Life on Land
Goal 16: Peace, Justice and Strong Institutions
**Goal 17: Partnership for the Goals**

* The goals deemed relevant by the authors and discussed in the context of AMR are presented in **boldface**.

## Data Availability

Not applicable.
